# Effect of a primary health-care-based controlled trial for cardiorespiratory fitness in refugee women

**DOI:** 10.1186/1471-2296-11-55

**Published:** 2010-08-02

**Authors:** Jan Sundquist, Maria Hagströmer, Sven-Erik Johansson, Kristina Sundquist

**Affiliations:** 1 Center for Primary Health Care Research, CRC, Lund University, Malmö, Sweden; 2 Department of Neurobiology, Care Sciences and Society, Division of Physiotherapy, Karolinska Institutet, Stockholm, Sweden

## Abstract

**Background:**

Refugee women have a high risk of coronary heart disease with low physical activity as one possible mediator. Furthermore, cultural and environmental barriers to increasing physical activity have been demonstrated. The aim of the study was to evaluate the combined effect of an approximate 6-month primary health care- and community-based exercise intervention versus an individual written prescription for exercise on objectively assessed cardiorespiratory fitness in low-active refugee women.

**Methods:**

A controlled clinical trial, named "Support for Increased Physical Activity", was executed among 243 refugee women recruited between November 2006 and April 2008 from two deprived geographic areas in southern Stockholm, Sweden. One geographic area provided the intervention group and the other area the control group. The control group was on a higher activity level at both baseline and follow-up, which was taken into consideration in the analysis by applying statistical models that accounted for this. *Relative aerobic capacity *and *fitness level *were assessed as the two main outcome measures.

**Results:**

The intervention group increased their *relative aerobic capacity *and the percentage with an acceptable *fitness level *(relative aerobic capacity > 23 O_2_ml·kg·min^-1^) to a greater extent than the control group between baseline and the 6-month follow-up, after adjusting for possible confounders (*P *= 0.020).

**Conclusions:**

A combined primary health-care and community-based exercise programme (involving non-profit organizations) can be an effective strategy to increase cardiorespiratory fitness among low-active refugee women.

**Trial Registration:**

ClinicalTrials.gov ID: NCT00747942

## Background

Demographic and economic changes have resulted in a more diverse population with greater social disparities in Sweden during the last few decades because of the large influx of refugees mainly from Latin America in the 1970 s, Iran in the 1980 s, Bosnia and the Balkans in the 1990 s, and Iraq and the Middle East in the 2000 s. Refugee status has previously been defined by the United Nations as a person who is outside the protection of his or hers country owing to a well-founded fear of being persecuted for reasons of race, religion, nationality, membership of a particular social group, or political opinion [1]. The concept of a refugee also includes persons who have fled war or other violence in their own country.

The large influx of refugees has changed Swedish society. A 2005 article in the *New York Times Magazine *provided data documenting the growing ethnic minority and socioeconomic segregation in urban and suburban Sweden [2].

Many of these refugees have an increased risk of coronary heart disease (CHD) in both men and women. Female refugees born in Turkey, Iran, and Iraq and settled in Sweden had an age-adjusted excess risk of CHD compared with Swedish-born controls ranging from 110% to 130% [3]. In spite of the well-known positive effects of physical activity on CHD, refugee women have low levels of physical activity [4-7]. Female refugees also have increased risks of overweight [8]. Furthermore, a report from the Swedish National Institute of Public Health showed that refugee women from Iran, Turkey, and Chile were less physically active than their Swedish counterparts. Among the Turkish-born women, only 1 out of 5 exercised regularly compared with 3 out of 5 among Swedish-born women [9]. A recent study using the International Physical Activity Questionnaire (IPAQ-long version) found that women from Iraq were less likely to report higher levels of physical activity than Swedish-born women after accounting for age, education, and smoking [10].

Although it is convenient to assess changes in physical activity with questionnaires, cardiovascular fitness is a more reliable and objective measure. For example, data from the longitudinal CARDIA study showed that a change in individual cardiorespiratory fitness is strongly associated with a corresponding change in habitual energy expenditure and leisure time physical activity [11]. Furthermore, it has been suggested that data on changes in fitness from one time point to another might be a better marker of change in physical activity than a change in physical activity assessed by questionnaire [12,13]. However, few studies have assessed cardiorespiratory fitness instead of physical activity in studies of refugees. For example, the Rockport Fitness Walk Test showed that a high percentage of the refugee Somali women had an estimated VO_2_max below the 50^th ^percentile and were also obese [14].

Physical inactivity and obesity are potential mediators of accelerating arteriosclerosis and the development of CHD seen among refugee women. According to the World Health Organization (WHO), it is important to target these risk factors in order to decrease the global burden of CHD. In addition, previous research has shown that the largest increase in physical activity levels can be found among the least active at baseline; minority groups were, however, not included in that study [15]. Therefore, it is important to use appropriate activities to target low active female refugees. To address this gap in the literature, our research group conducted a qualitative study of a group of refugee women in Sweden that emphasized that it is easier to do things in a group than on one's own and if appropriate activities are arranged, levels of physical activity and exercise might increase among refugee women [16]. The findings from that study provided in part a basis for the present intervention study.

Both individual and environmental factors are associated with physical activity in minority women. For example, a qualitative study from the United States showed that a lack of time due to caregiving duties, health worries, and lack of motivation were important barriers to women from ethnic minorities and refugee women becoming more physically active [17]. The women also felt that housekeeping and caregiving duties provided sufficient physical activity during the working day. In addition, a survey of four ethnic minorities in the US showed that women who had poor self-reported health, a low educational status, and reported a lack of energy, older age, a lack of hills in their neighbourhood, and only occasionally reported observing others exercising in their neighbourhood were exposed to significant risk for inactive lifestyles [18].

The real life situation of many refugee women in Sweden, such as low socioeconomic status, low educational status, and residing in a deprived and unsafe neighbourhood, has been associated with low levels of physical activity and exercise [19]. In this context, the present study is one of the first exercise intervention studies to recruit low-active refugee women in primary health care settings located in deprived areas. Another novel contribution of the present study is its focus on refugees from more than one country, which has the potential to be far more efficient than developing interventions for immigrants from only one country. Previous intervention studies have often focused on single immigrant groups [20,21].

The purpose of the present study was to investigate whether appropriate physical activities and social support can increase cardiorespiratory fitness in refugee women in Sweden. This purpose was implemented by evaluating the effect of an approximate 6-month primary health-care- and community-based exercise intervention versus an individual written prescription for exercise on objectively assessed cardiorespiratory fitness in low-active and overweight refugee women.

## Methods

### Design

This controlled clinical trial was named Support for Increased Physical Activity (*Stöd till Aktivare Motion *or *STAM *in Swedish).

### Setting

The refugee women were recruited between November 2006 and April 2008 from two deprived but distant geographic areas in southern Stockholm. We applied the widely used Swedish deprivation index, the Care Need Index (CNI), to identify the two deprived and immigrant-dense geographic areas (including large populations of Middle East refugees). The CNI scores ranged from-76.4 (most affluent neighbourhood) to 53.5 (most deprived neighbourhood) [22]. The CNI score in the two geographic areas was approximately 50, indicating a high level of deprivation in both areas. One geographic area provided the intervention group and the other area the control group. The rationale for using two distant geographic areas in Stockholm was because many refugee women live close to one another in such areas and we wished to minimize possible interactions between participants in the intervention and the control group.

### Subjects

Eligible for inclusion were women aged 25 through 64 who were first-generation refugees in Sweden from either the Middle East or Latin America, were overweight and reported low levels of physical activity during their leisure time (according to a validated question from the Swedish Annual Level of Living Survey, performed annually by the Swedish government-owned statistics bureau, see below) [10,23]. We excluded women with pre-existing ill-health conditions, which was based on the following question from the Swedish Annual Level of Living Survey: "Do you suffer from any long-standing illness, after-effects from an accident, disability, or other ailment of at least 3 months duration?" Exclusion criteria were a self-reported history of ischaemic heart disease, stroke, type 2 diabetes mellitus, and/or a disability that interfered with their ability to participate in lifestyle-related physical activity.

We appealed to the community to obtain access to physical activity facilities for the intervention. Nine primary health care centres located in the two immigrant-dense areas were partly used in the recruitment processes. This process included information brochures in the waiting room but the women were also informed about the study by the personnel at the primary health care centres. We also recruited women through local advertising and via community organizations such as women's associations, child welfare centres, pre-schools, churches/mosques and courses in Swedish for immigrants.

The women who were willing to participate filled in a first screening protocol. All women who filled in the screening protocol were contacted by research staff who could speak their native language. The participants were further informed about the study and those who agreed to participate and were eligible for inclusion were enrolled for a baseline assessment. Eligible for inclusion included 131 women in the intervention group and 112 women in the control group. All written material (flyers, questionnaires) was translated to Spanish or Arabic by two independent professional translators and backwards translated by two other professional translators.

In total three questions were included in the screening protocol to assess physical activity, overweight/obesity, and pre-existing ill health. Physical activity was assessed by asking the participants how often they exercise during their leisure-time. The response alternatives were the following: (1) I get practically no exercise at all, (2) I exercise occasionally, (3) I exercise regularly, about once a week, (4) I exercise regularly, about twice a week and (5) I exercise regularly, quite vigorously at least twice a week. Overweight and obesity were assessed on the basis of self-reported data on weight and height. Body mass index (BMI) was calculated as weight(kg)/height^2^(m) (according to WHO's recommendations) and comprised three categories: (1) normal weight (BMI < 25.0), (2) overweight (BMI 25.0-29.9) and (3) obesity (BMI ≥ 30).

Our intention was to include only those who were inactive (i.e. responded to alternative 1 or 2 in the physical activity question) and overweight/obese. However, around 10% in the intervention group and 50% in the control group were found to have a normal weight at the baseline measurements. They were not excluded from the study but we took BMI into account in the analyses.

Background information (such as education and language skills, see below) was assessed at baseline. Height, weight and cardiorespiratory fitness were assessed at baseline and at 6 months. In total, 212 women (*n *= 114 women in the intervention group and 98 women in the control group) were assessed at baseline. Most women came from the Middle East: 96% and 80% in the intervention and control group, respectively. The women mainly came from the following countries: Iraq, Syria, Turkey, Lebanon and Chile. The largest groups in the intervention group came from Syria (26%) and Lebanon (10%) and the largest groups in the control group came from Turkey (26%) and Iraq (24%).

### Intervention

It is generally presumed that the application of behavioural theories can increase the effectiveness of health behaviour change programmes [24]. A review of the exercise behaviour literature identifies theories applicable to physical activity, such as the social cognitive theory (SCT) and social ecological models. SCT is a theory of behaviour change that describes a dynamic ongoing process in which personal factors, environmental factors, and behaviour influence each other [25]. Three variables that are highly associated with exercise behaviour have been identified: self-efficacy, outcome-expectancy values, and self-regulation [25]. These variables were, however, not measured in the present study. Social ecological models posit that behaviour is shaped by both individual factors and environmental factors surrounding individuals in their daily life. Thus, it is more efficient to enhance the environment rather than to only change individuals, because enhancing one environment can have implications for many individuals. For the intervention, a combination of SCT and social ecological models was applied. SCT was used to strengthen self-efficacy and motivational support on the individual level [25]. A social ecological model was incorporated into this intervention both via the individually targeted intervention and via co-operation with local non-governmental organizations and community resources to establish a network of organizations that could provide participants with exercise opportunities and integrate their needs into the programme [26,27].

The intervention was culturally tailored to suit women from the Middle East and Latin America because its content was partly based on findings from a recent qualitative study from Sweden that included women from Chile, Iraq and Turkey [16]. The main finding of that study was that the women wanted to exercise but they needed help to start. They also wanted the exercise to be appropriate, arranged by the local community close to their homes and at times that did not conflict with their family duties. Moreover, the women emphasized that it is easier to do things in a group than by themselves. Although there are large disparities in the women's situation both within and between the Middle East and Latin America, the different countries included in the Middle East and Latin America probably represent cultures with strong paternalistic attitudes that could hinder women from taking part in physical activity programs. It is therefore likely that efforts to enable exercise are rather similar for these countries.

The approximate 6-month intervention included 28 weekly sessions with a one-month summer break after three months. Five additional sessions without a leader/instructor (see below) was given during the summer break. The women attended 16 sessions in average (range 1-32). An initial session of individual counselling with the purpose to strengthen self-efficacy, empowerment and personal motivation was given by a female physiotherapist who guided the women concerning how to start doing regular physical activities at a health-enhancing level and informed them about the benefits associated with physical activity. This initial session also addressed possible barriers to physical activity based on findings of a previous qualitative study, e.g. worries about palpitations, tiredness and sweating (15). The women received information about different levels of health-enhancing physical activity, such as moderate and vigorous physical activity and how they are related to perceived exertion [28,29]. The women were offered a possibility to choose between different types of group activities such as training in a gym, gymnastics and Nordic walking. Thus, the components in the group activities were treadmill walking, indoor bicycling and stair climbing, brisk walking and strengthening exercises. A new group started when at least six women had been recruited and each group consisted of 6-12 women. In total, we had 11 groups in the intervention group. The average number of participants in each group was 10.4. The groups were not separated by ethnicity. The physiotherapist was fluent in Arabic and Spanish translators were used, when needed. The same group of women met once a week to exercise together with a leader during the entire intervention in a space only for the women in whatever clothes they preferred. The female leaders/instructors were either the physiotherapist (see above) or a P.E. teacher. Both were employed and trained in the research team. The weekly sessions were arranged close to the women's homes and given at no cost. The women were free to come to the weekly sessions as much as they wanted. However, women who failed to turn up were contacted after two weeks and encouraged to return to the group activities. The group activities included one hour of physical training with integrated group counselling given by the trained leaders/instructors. The integrated group counselling addressed cultural barriers to physical activity in order to increase the women's empowerment and self-efficacy. The women were also encouraged to increase their overall physical activity level, i.e. walking for active transport and leisure or active play with their children.

The participants in the control group received an individual written prescription for exercise that was partly based on the results from the cardiorespiratory fitness together with an initial session of individual counselling. In general, the written prescription was based on the recommendation of regular health-enhancing physical activity, i.e. 30 minutes per day at a moderate intensity level [29,30]. The individual prescription for exercise was also given to the intervention group.

### Measures

#### Assessment of cardiorespiratory fitness

The Åstrand-Rhyming submaximal bicycle ergometer test was used to assess cardiorespiratory fitness [31]. The Monark bicycle ergometer, model 839E (Monark Exercise, Vansbro, Sweden) was used. It was calibrated with a 4-kg weight according to the manual. Bicycle seat height was adjusted so that the knee was almost fully extended at the lowest pedal position. Heart rate was recorded by telemetry directly linked to the bicycle ergometer. Participants were told to refrain from eating and smoking for 2 hours prior to testing. The participants were given an introduction to the test and the Borg rating of perceived exertion (RPE) scale [28]. The Borg RPE scale was used every minute to verify the heart rate and the resistance. The resistance was set to give a heart rate of about 120-150 (50-70% of heart rate range) and, for most subjects, 50, 75, or 100 watts was selected. The subjects were instructed to maintain the pedal rate at 50-60 revolutions per minute. One minute after starting the test, the heart rate was observed and the load was adjusted, if necessary, so that the heart rate would fall within the range in the final minutes of the test. If the heart rate difference between the 5th and 6th minutes was 5 beats or less, the test was terminated. If the difference was greater than 5 beats, the test was prolonged until the difference was not greater than 5 beats, but not more than a total of 12 minutes was allowed. Based upon the steady state, heart rate and the work load oxygen uptake (O_2 _l·min^-1^) were calculated by the bicycle computer, using the Åstrand-Rhyming nomogram (24). The data collectors in the present study were not blinded because we recruited the intervention and control groups from two different geographic areas.

Out of the 212 women who were assessed at baseline, 179 (84%) had at least one measurement either at baseline or at the six-month follow-up and they were included in the analysis (n = 91 in the intervention group and n = 88 in the control group). Most of the 33 excluded women were either not able to perform the test due to poor bicycling skills, or not able to bike at the correct speed, or had inadequate muscular strength to perform the test. A standardized walking test might have been an option; still, we believe that many of the women had too low a capacity to fit within any standardized protocol. The 33 excluded women did not differ with regard to age or BMI from those included.

To sum up, 243 women (131 women in the intervention group and 112 women in the control group) showed an initial interest to take part in the study and were considered eligible. Of these women, 212 came to the baseline assessments (114 women in the intervention group and 98 women in the control group) and 179 had at least one measurement either at baseline or at the six-month follow-up (91 women in the intervention group and 88 women in the control group). All women in the intervention group took part in at least one session with a leader/instructor.

#### Outcome variables

Two outcome variables were used. The first was *relative aerobic capacity*, defined as oxygen uptake per kilo body weight (O_2_ml·kg·min^-1^). The second outcome variable, *fitness level*, was dichotomized at a relative aerobic capacity larger than or equal to 23 O_2_ml·kg·min^-1 ^(1) and (0) otherwise. According to Blair, a relative aerobic capacity below 23 O_2_ml·kg·min^-1 ^is regarded as a high risk value for cardiovascular disease in women [32].

#### Independent variables

Self-reported *age *was categorized into 3 groups based upon tertiles of the data, 24-39, 40-44, and 45-64 years.

Height was measured to the nearest 0.5 cm using a stadiometer (Seca 214), and weight was measured using a bioimpedance analysis (8-contact electrode system; Tanita BC-418) to the nearest 0.1 kg, subtracting 2.0 kg for clothes. *BMI *(kg·m^-2^) was calculated from the objectively measured height and weight. BMI is categorized into 3 groups by the WHO (≤ 24.9 kg·m^-2^, normal- and underweight; 25-29.9 kg·m^-2^, overweight, and ≥ 30 kg·m^-2^, obesity [33]. In the present analyses, BMI was dichotomized at ≥ 30 kg·m^-2 ^(1) and otherwise as (0).

*Educational status *was classified into three categories: less than 10 years, 10-12 years, and more than 12 years.

*Employment status *was dichotomized as being employed or unemployed. Unemployed also included those on sick- or parental leave, retirees, students, and housewives.

*Acculturation *was assessed on the basis of language skills. The acculturation variable has been used previously by the Swedish government-owned statistics bureau to study integration and health in a random sample of Swedish immigrants from Chile, Iran, Poland and Turkey.

The participants in the present study answered to the following questions concerning: (1) knowledge of how to appeal against authorities, (2) ability to understand news reports, (3) speaking Swedish at meetings, (4) communicating with authorities over the telephone, (5) reading books in Swedish, and (6) being able to complete a written application for employment. The 1st question had two response alternatives (yes or no). The other five questions had four possible response alternatives, with 1 characterizing the highest degree and 4 the lowest degree of knowledge in Swedish. The answers to each question were dichotomized as one point for alternatives 1 and 2 (or 1 for question 1) and with zero points for alternatives 3 and 4 (or 2 for question 1). The dichotomy variables were summed up and categorized at two levels, with a low level of knowledge of Swedish/acculturation if the score was less than 4 and a high level of knowledge of Swedish/acculturation for a score of 4 or more.

*Years in Sweden *was assessed as a continuous variable.

### Statistical analysis

Baseline characteristics are shown as the median and interquartile range (IQR) for continuous data and number (*n*) and percentage (%) for categorical data (Table 1).

**Table 1:** Baseline characteristics (n =179^a)^).

	Intervention group *(n = 91)*	Control group *(n= 88)*	**P value**^**b)**^
*Continuous data*	**Median (IQR**^**c)**^**)**	**Median (IQR**^**c)**^**)**	
Age (years)	43 (11)	41 (11)	0.33
Height (cm)	156 (7)	158 (8)	0.26
Weight (kg)	76 (20)	68 (22)	0.002
BMI (kg/m^2^)	31 (8)	28 (9)	0.001
Years in Sweden	7 (15)	18 (16)	0.009
*Categorical data*	**n (%)**	**n (%)**	
Age			
18-39	43 (43)	37 (42)	0.94
40-54	49 (49)	43 (49)	
55-	7 (8)	8 (9)	
BMI category			
Normal weight (< 25 kg/m^2^)	9 (10)	24 (27)	0.008
Overweight	34 (37)	31 (35)	
Obese (≥ 30 kg/m^2^)	48 (53)	33 (38)	
Education			
< 10 years	49 (54)	36 (47)	0.65
10-12 years	18 (20)	19 (25)	
> 12 years	24 (26)	21 (28)	
Employed	26 (29)	36 (44)	0.03
Smokers	27 (30)	17 (19)	0.0001
Acculturation			
High level of knowledge of Swedish	20 (24)	40 (53)	0.0001
**Cardiorespiratory *fitness (outcomes)***	**Median (IQR)**	**Median (IQR)**	
Relative aerobic capacity (O_2_ml·kg·min^-1^)	23 (10)	32 (13)	0.0001
	**n (%)**	**n (%)**	
Fitness level ≥ 23 O_2_ml·kg·min^-1 ^(%)	49 (54)	73 (83)	0.0001

(n = 179^*a)*^)

^a^At least one measurement either at baseline or at 6-month follow-up of relative aerobic capacity.

^b^Wilcoxon rank sum test for continuous data and Chi-square test for categorical data.

^c^Interquartile range (IQR)

Baseline characteristics were compared for the 2 groups using the non-parametric Wilcoxon rank sum test for continuous variables due to non-normality and the chi-square test for categorized variables. A *P *value of less than or equal to 0.05 was considered significant.

We investigated the following possible confounders in both main analyses: age, BMI, education, employment status, and acculturation. However, only BMI and age were statistically significant and were therefore adjusted for in the final models.

Using a 2 (treatment) × 2 (time: baseline and 6-month follow-up) design, we analysed the treatment effect in relative aerobic capacity by applying a mixed linear model with random intercepts and slopes. Mixed model analyses allowed inclusion of subjects with missing data and differences between groups at baseline [34]. The results are shown as least square means and β-coefficients with 95% confidence intervals (CIs), adjusted for BMI and age.

We used a generalized estimating equations (GEE) model (with an exchangeable correlation matrix and robust standard errors) when analysing the dichotomized outcome, fitness level [35]. The results are shown as odds ratios (ORs) with 95% confidence intervals (CIs), adjusted for BMI and age.

The statistical package used was STATA Release 10 [36].

### Power calculation

To find a difference of 3 units in Δ for relative aerobic capacity (O_2_ml·kg·min^-1^) between groups with a power of 90% and a significance level α = 0.05 (based upon data from a pilot study with a mean of 24 O_2_ml·kg·min^-1 ^and a standard deviation of 7 and a correlation of 0.7 between occasions), 69 individuals per group were needed. An increase of 3 units for a person with very low aerobic capacity is important from a cardiovascular health perspective [37]. We reckoned with a drop-out rate of 30%, resulting in an estimated 90 women per group.

### Informed consent and ethical approval

The written informed consent of the participants was obtained. Participation was voluntary and the women had the option to terminate their participation at any time. The study was approved by the Regional Ethics Committee of the Karolinska Institute, Huddinge, Sweden (Reference 2005/1217-31).

## Results

The characteristics of the study population are shown by group in Table 1 for those women with at least one measurement either at baseline or at 6-month follow-up of relative aerobic capacity. The intervention group had lived a shorter time in Sweden, had a higher prevalence of obesity and smoking, a lower employment rate, and poorer language skills than the control group (*P *< 0.05). The control group had a significantly better *relative aerobic capacity *and a higher percentage with an acceptable *fitness level *(*relative aerobic capacity *> 23 O_2_ml·kg·min^-1^) at baseline than the intervention group.

We found that age and BMI significantly influenced the mixed model for *relative aerobic capacity *as outcome, while educational level, employment status, and acculturation did not. Table 2 shows the adjusted (for age and BMI) least square means with 95% confidence intervals for relative aerobic capacity based on a mixed model (with an exchangeable variance-covariance structure). We also found a significant interaction between treatment and test occasion (*P *= 0.02). This indicates that there is a treatment effect, i.e. the intervention group improved their *relative aerobic capacity *significantly more than the control group.

**Table 2:** Least square means (95% confidence interval) for treatment effect^a) ^of relative aerobic capacity.

Outcome	Group	Baseline	6-month	P value
Relative aerobic capacity (O_2_ml·kg·min^-1^)	Intervention	24.5 (23.2-25.9)	29.3 (27.4-31.2)	(trt × time) = 0.020
	Control	30.5 (29.2-31.9)	32.5 (30.6-34.3)	

^a^Treatment effect = the interaction between group and time (trt*time) in relative aerobic capacity, adjusted for age and BMI.

The β-coefficients with 95% confidence intervals are shown in Table 3. The intervention group showed a significantly (*P *= 0.00001) larger increase over the 6-month follow-up in relative aerobic capacity than the control group (*P *= 0.02). However, the control group had a higher relative aerobic capacity than the intervention group at both baseline (*P *= 0.0001) and the 6-month follow-up (*P *= 0.01).

**Table 3:** Regression coefficients (β) and odds ratio (OR) with 95% confidence interval (CI) for the outcomes^a)^.

Outcome	Group	Baseline	6-month	P value
		**β(CI)**	**β(CI)**	
Relative aerobic capacity (O_2_ml·kg·min^-1^)	Intervention	0 (reference)	4.8 (3.0-6.5)	(trt × time) = 0.02
	Control	6.0 (4.1-8.0)	7.9 (5.7-10.2)	

		**OR (CI)**	**OR (CI)**	

Fitness level > 23 O_2_ml·kg·min^-1^	Intervention	1 (reference)	3.7 (1.8-7.8)	(trt × time) = 0.02
	Control	5.0 (2.3-10.9)	5.6 (2.4-13.2)	

^a ^Relative aerobic capacity (O_2_ml·kg·min^-1^) and fitness level ≥ 23 O_2_ml·kg·min^-1 ^; the models are shown as the interaction between treatment and time (trt*time), adjusted for age and BMI.

The GEE model showed results similar to those of the mixed model with a significant interaction between treatment and time (*P *= 0.02), also including age and BMI, when analysing the dichotomized outcome *fitness level *(*relative aerobic capacity *> 23 O_2_ml·kg·min^-1^). Table 3 shows that the intervention group increased the odds from baseline to follow-up to a greater extent than the control group (from OR = 1 at baseline to OR = 3.7, 95% CI = 1.8-7.8 at follow-up). The control group was almost unchanged (OR = 5.0; 95% CI = 2.3-10.9 and OR = 5.6; 95% CI = 2.4-13.2, respectively). However, the control group was on a higher level at both baseline and follow-up.

## Discussion

The main finding of this controlled clinical trial was that the intervention group increased their *relative aerobic capacity *and the percentage with an acceptable *fitness level *(*relative aerobic capacity *> 23 O_2_ml·kg·min^-1^) to a greater extent than the control group from baseline to the 6-month follow-up, after adjustments for age and BMI. However, the control group was on a higher level at both baseline and follow-up, which was taken into consideration by employing a mixed linear model with random intercepts and slopes and, for *fitness level*, a GEE model.

The finding that a combined primary health care- and community-based exercise intervention increased the relative aerobic capacity and the percentage with an acceptable fitness level is important because such an intervention may have the potential to decrease lifestyle-related disease risks such as coronary heart disease, cancer, and diabetes mellitus in low-active refugee women. Cardiorespiratory fitness, which we measured as maximum oxygen consumption (VO_2_max), determined during submaximal exercise testing, has been shown to provide a good estimate of cardiorespiratory capacity, which is an independent marker of the early atherosclerotic cardiovascular diseases [38] and a significant predictor of all-cause mortality among women with impaired fasting glucose or undiagnosed diabetes mellitus [39].

This study used a novel design that combined primary health care and community efforts to increase fitness among refugee women in deprived neighbourhoods. We used primary health care facilities and other community resources to identify, recruit, and examine not regularly physically active women and appealed to the community to access facilities for the intervention. Primary health care is an important setting for interventions because, in a country like Sweden, nearly the entire population visits a primary health care facility during a 3-year period. Therefore, a physical activity referral scheme has been introduced in primary health care to improve physical activity levels. Such a scheme carried out in routine health care settings does indeed increase physical activity levels [15]. Furthermore, a recently published study from a primary care setting in northern Sweden found that intensive lifestyle modification among high-risk individuals favourably impacted aerobic fitness [40]. These studies did, however, not include foreign-born patients. Although physical inactivity and obesity are prevalent among foreign-born people, few investigators include them in intervention studies because this increases the complexity of the study with the use of interpreters and the difficulties in managing participants from different cultures.

We want to stress that cultural factors depending on country of birth influence physical activity [16]. For example, Nakamura described the influence of Muslim culture on physical activity patterns among women and found that a lack of prerequisites for participation in the form of gender segregation, the lack of a flexible and modest dress code or controlled access to their physical activity space rather than their faith prevented the women from participating in physical activity programmes [41]. This supports the rationale and design of this study in which we provided a space only for the women to do their exercise in whatever clothes they preferred.

Our findings support the need for a structured programme, such as that developed in our study, to produce an effect in high-risk groups. In the present study we have included individual and integrated group counselling to address cultural barriers to physical activity as well as access to community physical activity centres where the women obtained support for behavioural changes and participation in group activity classes. In contrast to our findings, an American study evaluated the effects of a tailored physician-delivered intervention (individualized advice) in comparison with standard care in a primary care setting of sedentary ethnic minority and low-income women [42]. Their results suggested that a physician-delivered intervention has limited effectiveness for increasing physical activity in this high-risk population.

A protocol for an ongoing randomized controlled trial in primary health care recently reported on the effectiveness of an exercise referral scheme in the short and long term among women from ethnic minority groups [43]. Several other studies have evaluated the effect of community-based lifestyle interventions in minority groups. For example, a community-based educational intervention in the US targeting high-risk ethnic minority women found that significant improvement was attained in most of the 28 secondary outcomes, but not in the primary outcomes (reducing obesity and increasing physical activity). They concluded that a heart disease prevention intervention built around a model of community engagement, advocacy, self-efficacy, resource knowledge, and health promotion in faith- and community-based organizations is successful in high-risk women [44]. Another study by Wellman et al. (2007) developed a community-based integrated nutrition and exercise programme designed for older Americans, with a majority of participants from minority groups [45]. The results showed that 75% of the respective participants made a significant advance of one or more nutrition and physical activity stages of change. This supports the use of community-based strategies or, as in our study, the combined primary health care and community-based approach.

One limitation in the design of the study is the use of a controlled clinical trial instead of a randomized controlled trial (RCT). Conducting a study in a real-life setting that can be implemented in the society at large is difficult. Although an RCT would have been optimal from a study design perspective, bias may be present in an RCT [46]. For example, the inclusion of randomized subjects from the same geographic area might affect the study results if subjects from the intervention and control groups interact. Therefore, we chose to include participants for the intervention and the control group from two similar but distant geographic areas in Stockholm. Another limitation is that the sample size did not allow for the assessment of the relative benefits of the different types of activities or whether women from a particular country preferred a certain type of activity. Although our measure of acculturation did not influence the models, it may have not been able to reflect the rather complex nature of acculturation. Acculturation is a broader concept than just language, although language skills have been shown to be a robust indicator of self-reported health and cardiovascular risk factors [47,48]. The programs were offered free of charge, which is not feasible in the long run. Therefore, it is unknown whether the women would continue to be active were there a charge. The intervention group had considerably lower relative aerobic capacity than the control group at baseline, and studies in exercise physiology have shown that it is easier to increase the aerobic capacity when the initial level is low [49]. Even though the mixed models took account of the fact that the two groups were different at baseline, we cannot rule out that this physiological mechanism explained parts of the treatment effect. There were also other differences between the intervention and control group at baseline, which the mixed models accounted for. Physical activity was assessed by asking the participants how often they exercise during their leisure-time. Other types of physical activity, such as domestic activities, were not assessed. Although implementation fidelity was not specifically measured, due care was taken in order to deliver the intervention as intended in the following dimensions: adherence, content, process, dose, quality of delivery and competence [50]. Finally, this intervention was evaluated after 6 months and any long-term effect remains to be evaluated. However, the results indicate a potential risk reduction for future cardiovascular disease in this high-risk group.

This study also has several strengths. One is that it was conducted using existing community institutions, such as primary health care centres, which means that the model can be readily implemented. There are challenges in recruiting minority groups to prevention programmes with the aim to reduce cardiovascular risk factors [51,52].

A recent systematic review was able to identify only 13 intervention studies among immigrants of which three focused on cardiovascular risk factors [21]. Most of these studies used churches or faith-based clinics in the recruitment process whereas the present study applied a much broader concept in the recruitment process, including nine primary health care centres, women's associations, child welfare centres, pre-schools, churches/mosques, courses in Swedish for immigrants and local advertising. This approach is in line with a previous study of strategies for recruiting minority women. That study employed a range of culturally appropriate strategies to recruit Hispanic women into a prospective cohort study of modifiable risk factors [51].

Another strength is the use of a group-based intervention to be used in a country with a large and diverse immigrant population, particularly from the Middle Eastern countries. Besides the fact that the women can support one another, it is also cost-effective for the community compared to programmes for individuals or single immigrant groups. Finally, this study was evaluated using an objective measure of fitness, not just the self-reported level of physical activity, which is subject to measuring errors [53].

## Conclusions

This controlled clinical trial showed that a combined primary-health-care and community-based exercise programme can constitute an effective strategy to increase fitness among low-active overweight refugee women who are at risk of future coronary heart disease.

## Competing interests

The authors declare that they have no competing interests.

## Authors' contributions

JS, KS and SEJ contributed to the design of the study. All authors contributed to the analysis and interpretation of data. MH drafted the first manuscript. All authors revised the manuscript for important intellectual content and gave their final approval of the version to be submitted.

## Pre-publication history

The pre-publication history for this paper can be accessed here:

http://www.biomedcentral.com/1471-2296/11/55/prepub
